# The Role of Phytoplankton and Sediment Microbial Community on Sr, U, Pu, and Am Behavior in Freshwater Lake Dryazlo

**DOI:** 10.3390/biology15090724

**Published:** 2026-05-02

**Authors:** Marina Popova, Vasiliy Riabov, Nadezhda Popova, Grigoriy Artemiev, Alexey Safonov

**Affiliations:** 1 V. Vernadsky Institute of Geochemistry and Analytical Chemistry, Russian Academy of Sciences, Kosygina Str. 19, 119991 Moscow, Russia; marbpop@gmail.com (M.P.); riabov.vasily.ucheba@gmail.com (V.R.); 2 A.N. Frumkin Institute of Physical Chemistry and Electrochemistry, Russian Academy of Sciences, Obrucheva Str. 40, 117342 Moscow, Russia; nm.popova.ipce.ras@gmail.com (N.P.); artemyev56@gmail.com (G.A.)

**Keywords:** strontium and actinides behavior, phytoplankton and bottom sediments microbial diversity, eutrophication, iron and sulfur microbial cycle

## Abstract

Radionuclide contamination of surface water bodies poses a significant environmental challenge, particularly for low-productivity dystrophic systems where natural self-purification capacity is limited. This study aimed to assess the potential of phytoplankton and bottom sediments as biogeochemical barriers for radionuclides. Laboratory modeling of ^90^Sr, ^233^U, ^239^Pu, and ^241^Am accumulation was conducted using samples of Lake Dryazlo (Tver Oblast) water and bottom sediments as a representative dystrophic model system. Sorption onto phytoplankton biomass over a single growing season was estimated at 1.89 × 10^4^, 5.41 × 10^4^, 6.64 × 10^4^, and 4.04 × 10^4^ Bq g^−1^ dry biomass for ^90^Sr, ^233^U, ^239^Pu, and ^241^Am, respectively. Actinide immobilization in bottom sediments depended on mineral composition and microbial community activity. Ammophos addition increased radionuclide removal from the liquid phase by 2–5-fold through enhanced phytoplankton productivity, and promoted actinide fixation via phosphate mineral phase formation and stimulation of anaerobic sulfur- and iron-cycling bacteria. These results demonstrate a viable biogeochemical barrier approach applicable to the decommissioning of radioactive waste storage ponds and remediation of radionuclide-contaminated water bodies.

## Simple Summary

Laboratory modeling of ^90^Sr, ^233^U, ^239^Pu, and ^241^Am accumulation was conducted using samples of Lake Dryazlo (Tver Oblast) water and bottom sediments as a representative dystrophic model system. Sorption onto phytoplankton biomass over a single growing season was estimated at 1.89 × 10^4^, 5.41 × 10^4^, 6.64 × 10^4^, and 4.04 × 10^4^ Bq g^−1^ dry biomass for ^90^Sr, ^233^U, ^239^Pu, and ^241^Am, respectively. Actinide immobilization in bottom sediments depended on mineral composition and microbial community activity. Under high trophic conditions, the activity of microorganisms participating in the nitrogen and sulfur cycles results in more robust immobilization of radionuclides in bottom sediments, with their distribution coefficients increasing by an order of magnitude.

## 1. Introduction

Radionuclide contamination of aquatic ecosystems resulting from nuclear industry operations represents one of the most pressing environmental challenges. Notable examples of significant impacts on the biosphere include the discharge of radioactive waste into the Techa River and Lake Karachay at the Mayak Production Association (PA Mayak) and the Siberian Chemical Combine (SCC) [1], releases into the North Sea from the Sellafield reprocessing plant, United Kingdom [2], leakage from storage facilities at the Hanford Site, USA [3], and incidents at other nuclear installations. A substantial contribution to the radioactive contamination of water bodies has been made by the atmospheric dispersion of radionuclide-bearing aerosols during accident events, most notably the Kyshtym disaster at PA Mayak in 1957 and the Chernobyl Nuclear Power Plant accident in 1986 as well as nuclear weapons testing. The principal radionuclides contaminating freshwater systems vary depending on the source of contamination and include isotopes of ^90^Sr, ^137^Cs, ^60^Co, ^235–238^U, ^239^Pu, ^241^Am, and others [4,5], whereas marine environments are predominantly affected by ^90^Sr, ^137^Cs, ^144^Ce, ^91^Y, ^95^Nb, ^235–238^U, ^239^Pu and ^241^Am [6]. Furthermore, considering their long half-lives and complex speciation under different redox conditions, actinides present in the environment demand particular scrutiny.

It should be noted that the mobility and bioavailability of radionuclides within a water body are governed by a range of parameters, including its hydrological and wind-wave regime, bottom topography, depth, meteorological conditions, physicochemical characteristics (temperature regime, pH, Eh, salinity, etc.), as well as the taxonomic and functional diversity of the biota—most critically the microbial community and lower plants. Apart from phytoplankton and the microbial communities inhabiting bottom sediments, higher aquatic plants and benthic and benthonic organisms—including fish, mollusks, and other fauna—play a significant role in the accumulation of radionuclides in freshwater bodies [7]. The radioecological hazard posed by radionuclides in aquatic environments is primarily determined by their physicochemical speciation: their chemical form and state in solution (dissolved, as organic or mineral complexes, or associated with colloidal or pseudo-colloidal particles). Under certain conditions, favorable geochemical environments may develop within a water body that promote radionuclide immobilization in bottom sediments. This process proceeds via adsorption onto suspended particulate matter followed by gravitational settling to the sediment layer. Biogenic processes, including photosynthesis (which governs the oxygen regime of the water body), biosorption, biomineralization, bioaccumulation, and radionuclide assimilation by phytoplankton substantially enhance the intensity of self-purification. Phytoplankton, comprising microalgae and cyanobacteria, plays a pivotal role in radionuclide sedimentation, particularly during periods of intense bloom development in the summer season [8]. Active biomass growth generates a large sorption surface area [9,10], facilitating efficient radionuclide removal from the water column upon cell senescence and death. The accumulation of radionuclides by microalgae is further promoted by their capacity to produce phytochelatins—a family of macromolecules bearing diverse functional groups which enable cells to sequester trace elements required for growth or, under adverse conditions, to reduce cytoplasmic concentrations of toxic metals [11]. However, during seasonal transitions (winter and early spring) and shifts in dissolved oxygen saturation within the water column, radionuclide desorption may occur in shallow water bodies with low biological productivity, resulting in elevated radionuclide concentrations in the aqueous phase [12,13,14].

Thus, high biological productivity of water bodies leading to the formation of substantial organo-mineral bottom sediment layers constitutes an important factor ensuring both the intensity of self-purification and the reliable long-term immobilization of radionuclides within the active sediment layer [15,16]. One of the key mechanisms of radionuclide immobilization in bottom sediments is biomineralization, whereby the metabolic activity of anaerobic sulfur-cycling microorganisms promotes the formation of low-solubility precipitates such as pyrrhotite, troilite, and hydrotroilite [17]. The anaerobic iron-cycling microbial community, in turn, facilitates the formation of oxidized low-solubility ferrous phases, including ferrihydrite and other iron oxyhydroxides, which possess high sorption capacity for a broad range of radionuclides [18,19,20,21].

Since the primary focus of researchers has been directed toward the behavior of radionuclides in water bodies subject to anthropogenic influence, including those contaminated as a result of accidents or releases from industrial facilities [22,23,24,25,26], as well as natural or artificial reservoirs serving as repositories for radioactive waste [27,28] the majority of such studies have been conducted on phytoplankton communities and bottom sediments altered by technogenic factors. Investigations of radionuclide behavior in marine ecosystems [29,30,31] and large river systems are subject to considerable limitations owing to the substantial contribution of hydrogeological factors, such as currents and significant water depth, among others. For this reason, studies of small enclosed water bodies of shallow depth that have not been subjected to technogenic impact are of particular importance in radioecological research concerned with the distribution of radionuclides between the aqueous phase and bottom sediments. Work with such systems may yield more detailed information on the role of physicochemical, geochemical, and biogeochemical factors governing radionuclide behavior, and may subsequently serve as a basis for modeling these processes in larger water bodies. A representative example of a dystrophic lake that allows for the assessment of the role of eutrophication in radionuclide behavior is Lake Dryazlo, located in the Nelidovsky District of Tver Oblast, Russia. Currently, no industrial or agricultural activities are conducted in the area surrounding the lake; therefore, it can be regarded as a typical lake for the European part of Russia with minimal anthropogenic impact.

The aim of the present study is to model the accumulation of Sr, U, Pu, and Am by phytoplankton and the bottom sediments derived therefrom, under stimulation with various sources of biophilic elements, using Lake Dryazlo (Tver Oblast) as a model system—a dystrophic water body characterized by comparatively low biological productivity.

## 2. Materials and Methods

### 2.1. Study Site Description

**Figure 1:**
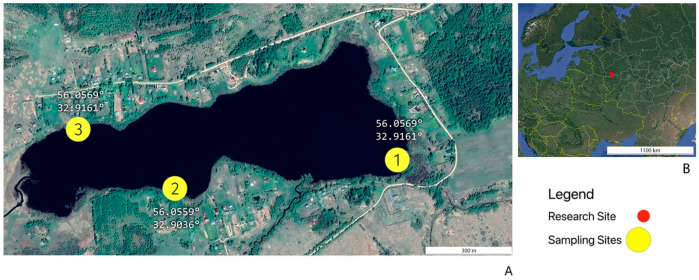
(**A**) View of the lake and water sampling location. Water samples (L1, L2, L3) and sediment samples (B1, B2, B3) were collected from each site; (**B**) Location of the lake Dryazlo.

Water and bottom sediment samples used in this study were collected from Lake Dryazlo (56°03′30″ N, 32°54′20″ E), located in the southwestern part of Tver Oblast (Figure 1). Lake Dryazlo is a glacial lake in origin, with a mean depth of 2.0–3.5 m. The surface area of the lake is 0.4 km^2^. It is fed by the inflow of the Luchesa and Tagoshcha rivers and has a low water exchange rate. According to the Köppen–Geiger climatic classification, the study area is situated within the humid continental (Dfb) climatic zone, characterized by humid and warm summers. Mean annual precipitation is approximately 650 mm. January is the coldest month, with a mean temperature of −9.5 °C, while July is the warmest, with a mean temperature of +17.6 °C.

### 2.2. Sampling

Sampling was conducted during the year 2020. For the analysis of chlorophyll and organic matter concentrations, 1.5 L water samples were collected from April to October from a boat positioned in the middle of the lake at a depth of 50 cm below the surface. Temperature and dissolved oxygen concentration were measured in situ by submerging the respective sensors. Water samples for chemical analysis were collected using a bathometer in June 2020 from sampling points L1–L3. Three replicate samples (1.5 L each) were collected from the water column at a depth of 50 cm below the surface under oxygen-free conditions. The samples were stored in a refrigerator at +4 °C until analysis. For iron content determination, samples were fixed with concentrated HNO_3_ to pH < 2. Bottom sediment samples were collected in July 2020. At each sampling point, three replicate samples were collected for mineralogical and elemental analyses. Samples for 16S rRNA gene amplicon sequencing were collected into sterile vials, fixed with ethanol, and subsequently stored at +4 °C until DNA extraction. Phytoplankton samples were collected during the period of active bloom development in July 2020. At the time of sampling, water temperatures ranged from 20 to 23 °C, and pH values ranged from 7.35 to 7.45. The oxidation-reduction potential (Eh) was 341 mV in the near-surface water column (5 cm depth) and 151 mV at the sampling depth (50 cm). Water samples for laboratory experiments were collected in early June, prior to the onset of the active bloom period, using a bathometer at a depth of 0.5 m. The samples were collected into sterile 1 L vials and stored in a refrigerator at +4 °C. Bottom sediment samples for laboratory experiments were also collected into sterile vials in early June and stored at +4 °C until the start of the experiments.

### 2.3. Experimental Design

#### 2.3.1. Assessment of Mineral Additive Effects on Phytoplankton Biomass Growth

**Figure A1:**
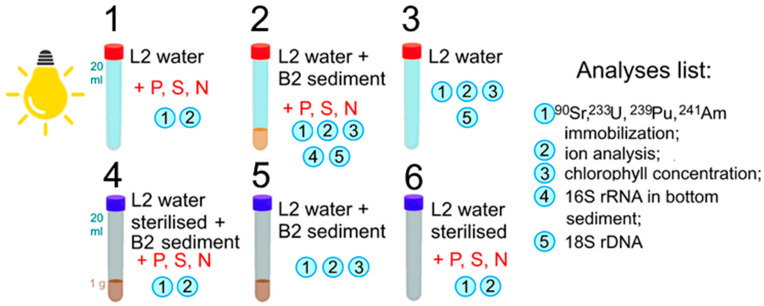
Laboratory experimental design.

Twenty-milliliter vials containing lake water samples (L2) and sediment (B2) samples were used for the laboratory experiments. The duration of the laboratory experiment was 45 days (until complete sedimentation of cells to the bottom sediment layer) at room temperature (21–23 °C). Laboratory modeling (Figure A1, see Appendix A) involved a series of modifications applied to water and bottom sediment samples, aimed at assessing the effect of trophic conditions on radionuclide behavior through a single addition of a mineral additive (ammophos, 100 mg/L; Na_2_SO_4_, 100 mg/L):

- 1—eutrophic conditions, lake water sample L2 (under constant illumination at 2000 K);
- 2—eutrophic conditions, bottom sediment sample B2 and water L2 (under constant illumination at 2000 K);
- 3—dystrophic conditions, lake water sample L2 (under constant illumination at 2000 K);
- 4—eutrophic conditions, bottom sediment sample B2 and water L2;
- 5—lake bottom sediment sample B2 and water L2;
- 6—eutrophic conditions, sterile lake water sample L2

#### 2.3.2. Evaluation of Radionuclide Immobilization Efficiency During Phytoplankton Development

Experiments were conducted in polypropylene vials containing 50 mL of lake water, to which radionuclide stock solutions were added. Nitric acid solutions of the following radionuclides were used (specific activities in the sample given in mol/L and Bq/L, respectively): ^90^Sr—1.4 × 10^−10^ mol/L (6.5 × 10^4^ Bq/L); ^233^U (UO_2_^2+^)—4.8 × 10^−7^ mol/L (4.0 × 10^4^ Bq/L); ^239^Pu—1.8 × 10^−8^ mol/L (1.0 × 10^4^ Bq/L); ^241^Am—1.0 × 10^−9^ mol/L (3.0 × 10^4^ Bq/L). The radionuclides were obtained from JSC “All Regional Association ‘Izotop’” (Moscow, Russia).

Sorption experiments were conducted in the dark at 20 °C using air-dried bottom sediments at a solid-to-liquid ratio of 1:20. Water samples collected from the corresponding sampling sites and sterilized by filtration through a 220 nm pore-size membrane filter were used as the liquid phase. The duration of the experiment was up to 10 days, until quasi-equilibrium conditions were attained, under continuous stirring.

Aliquots of the liquid phase were collected at defined time intervals for radionuclide analysis. The degree of radionuclide sorption (S, %) and the corresponding distribution coefficient (K_d_) were determined from the decrease in activity in solution according to the following expression:

(1) $$K_{d}=\frac{C_{sp}}{C_{lp}}\cdot \frac{V}{m}$$

where C_sp_ is the radionuclide concentration in the solid phase; C_lp_ is the radionuclide concentration in the liquid phase; V is the volume of the liquid phase (cm^3^); and m is the mass of the solid phase (g).

To assess the binding strength of sorbed radionuclides, a single-stage desorption experiment was performed following air-drying of the sediment samples, using the same lake water as the desorption medium.

Speciation of sorbed radionuclides was performed for the bottom sediments samples obtained after sorption experiments using the modified Tessier sequential extraction technique listed in Table A1 (see Appendix A). All stages were performed under aerobic conditions with constant stirring for 2 h, a temperature of 298 K, and the S/L ratio of 1/40. The specific activity of solutions was determined similar to the technique used at the sorption stage. The residual technetium content was calculated based on data obtained from five sequential washing steps.

### 2.4. Analytical Methods

Temperature and dissolved oxygen were measured with a MARK-302T («VZOR» LLC, Nizhniy Novgorod, Russia) analyzer using a flow-immersion amperometric probe.

X-ray diffraction (XRD) analysis was performed on a Panalytical Aeris (Panalytical, Malvern, UK) powder X-ray diffractometer (Cu Kα anode at 40 kV and 15 mA) at the Shared Use Center, Institute of Physical Chemistry and Electrochemistry, Russian Academy of Sciences. Samples were dried and ground in a corundum mortar to a fine powder. Data were collected over a 2θ range of 2° to 65° in 0.002° increments using a ¼ rad slit. The results were interpreted using the HighScore Plus software ver. 2020 (https://www.malvernpanalytical.com/en/products/category/software/x-ray-diffraction-software/highscore-with-plus-option/, accessed on 15 January 2025) with the PDF2 database (https://www.icdd.com/pdf-2/, accessed on 15 January 2025). The total clay fraction content was estimated using the Rietveld method, and individual clay minerals (montmorillonite, illite, and kaolinite) were identified by characteristic peaks in oriented specimens, following the Clay Mineral Identification Flow Diagram (https://pubs.usgs.gov/of/2001/of01-041/htmldocs/flow/index.htm, accessed on 20 January 2025).

Scanning electron microscopy (SEM) was carried out using a TESCAN MIRA3 scanning electron microscope (TESCAN, Brno, Czechia) at the Joint Use Center, Vernadsky Institute of Geochemistry and Analytical Chemistry, Russian Academy of Sciences. Prior to analysis, samples were removed from the liquid medium and dried at room temperature in a nitrogen glove box to constant weight. Specimens were mounted on aluminum stubs using electrically conductive tape and subjected to vacuum carbon deposition on a Q150T E Plus coater; vacuum 4 × 10^−3^ Pa, current 50 A). Samples were examined in both secondary electron (SE) and backscattered electron (BSE) modes at an accelerating voltage of 20 kV.

Elemental concentrations in water samples were determined immediately after collection and filtration through a 0.45 µm glass membrane filter by Inductively Coupled Plasma Mass Spectrometry (ICP-MS) using an X Series2 instrument (Thermo Fisher Scientific, Waltham, MA, USA) and by Inductively Coupled Plasma Atomic Emission Spectroscopy (ICP-AES) using an iCAP 6500 instrument (Thermo Fisher Scientific, Waltham, MA, USA).

Anion and cation concentrations were determined by capillary gel electrophoresis using a Capel-105M system (LUMEX Instruments, Saint Petersburg, Russia).

Eh and pH measurements were performed using an ANION-4100 pH meter/ionometer (ANION LCC, Novosibirsk, Russia, http://www.anion.nsk.su/, accessed on 20 January 2025) with a combined Ag/AgCl reference electrode.

Radionuclide content was determined by the liquid scintillation method using the Tri-Carb-3180 TR/SL radiometer (Perkin-Elmer, Shelton, CT, USA) in scintillation cocktail (OptiphaseHisafe 3, “PerkinElmer,” Drachten, The Netherlands). Detection limits (Bq/L) 0.4 for ^90^Sr, 0.04 for ^239^Pu, 0.02, for ^233^U, 0.06 for ^241^Am.

Phytoplankton biomass was quantified by two complementary methods: gravimetric determination of dry cell weight and spectrophotometric analysis of chlorophyll concentration. For dry weight determination, biomass was separated from the culture medium by filtration through Millipore membrane filters (pore diameter 0.22 µm). Filters were brought to constant weight in an oven at 80 °C, and 10 mL of culture suspension of known optical density was then filtered under vacuum. The filters were dried again to constant weight, and the dry cell mass (mg/mL) was calculated from the difference in filter weights. For centrifugation-based biomass separation, an OPN-8m centrifuge was operated at a rotor speed of 6000 rpm.

DNA was extracted from phytoplankton biomass and bottom sediment samples using the ZymoBIOMICS™ DNA Miniprep Kit (Zymo Research, Irvine, CA, USA) according to the manufacturer’s protocol. Partial 18S rDNA (435 bp, including the highly variable V4 region) gene was amplified using primers D512for and D978rev from Zimmermann et al. [32]. Variable regions V3–V4 of the 16S rRNA gene were selected for library preparation and amplification. Real-time PCR amplification was performed on a CFX96 Touch instrument (Bio-Rad, Hercules, CA, USA) using the qPCRmix-HS SYBR reaction mixture (Evrogen, Moscow, Russia). High-throughput sequencing was conducted on a MiSeq platform (Illumina, San Diego, CA, USA) using the MiSeq Reagent Kit v2 (Illumina, San Diego, CA, USA) (500 cycles).

Amplification conditions for 18S rDNA gene were as follows: initial denaturation for 5 min at 95 °C followed by 35 cycles of 30 s denaturation at 94 °C, 30 s annealing at 52 °C, and 50 s extension at 72 °C, with the final extension for 10 min at 72 °C. PCR products were visualized by horizontal electrophoresis in 1.0% agarose gel stained with SYBRTM Safe (Life Technologies, Carlsbad, CA, USA). The products were purified with a mixture of FastAP, 10× FastAP Buffer, Exonuclease I (Thermo Fisher Scientific, Waltham, MA USA), and water. The sequencing was performed using a Genetic Analyzer 3500 instrument (Applied Biosystems, Foster City, CA USA).

Bacterial community libraries were created using the online SILVA resource (https://www.arb-silva.de/ngs/, accessed on 20 March 2025). Raw reads were processed and assembled as previously described [33]. Contigs were binned and refined, MAGs were reassembled by BASALT v1.1.0 [34]. MAGs were analyzed by METABOLIC v4.0 [35]. Adapters and primers were trimmed with cutadapt v2.8 [36], quality filtering was done by Trimmomatic v0.36 [37]. Demultiplexing was done by deML v1.1.4 [38]. Chimeric sequences were removed and the amplicon sequence variant (ASV) table was constructed using Dada2 v1.26.0 package and the SILVA 138.2 database [39]. Sequencing data obtained were processed using the online platform (https://www.microbiomeanalyst.ca/, accessed on 20 March 2025) marker data profiling and shotgun data profiling [40]. OTU lower 1% were excluded from further analyses. Following processing, taxonomic composition diagrams of the microbial community, a Venn diagram, and physicochemical profiling were generated using OriginPro ver 2024 (https://www.originlab.com/2024, accessed on 20 March 2025). Biodiversity indices were calculated using Past5 software (https://www.nhm.uio.no/english/research/resources/past/, accessed on 20 March 2025).

### 2.5. Geochemical Modeling

Geochemical modeling was performed using PHREEQC 2.18 (pH–REdox–EQuilibrium) software (https://phreeqc-interactive-alpha.software.informer.com/download/, accessed on 31 March 2025) to determine elemental speciation in solution and to calculate mineral saturation indices. The PHREEQC code is based on the numerical solution of systems of mass action law and mass balance equations. Saturation indices (SI) for solid mineral phases of the radionuclides under investigation were evaluated during modeling. The saturation index SI is defined as the difference between the decimal logarithms of the ion activity product (IAP) of the i-th phase and the corresponding solubility constant:

SI = logIAP − logKs, (2)

where IAP is the ion activity product in solution and Ks is the equilibrium solubility constant of the mineral phase.

An SI value below zero indicates that the phase is undersaturated (prone to dissolution), whereas an SI value above zero indicates supersaturation and the likelihood of precipitation. Three thermodynamic databases were employed for modeling: llnl, PSINA, and NEA.

### 2.6. Statistics

Data statistical processing (average of chemical and mineralogical analysis results and laboratory experiment data across three replicates per sample) was carried out using the built-in tools of Origin ver. 2024. The results of the diversity analysis are based on a single run and are therefore descriptive in nature.

## 3. Results

### 3.1. Characterization of Water and Bottom Sediment Samples

#### 3.1.1. Ionic Composition of Water Samples

The concentrations of major ions in the water samples are presented in Table 1. Total dissolved solids did not exceed 400 mg/L. Dissolved organic matter ranged from 12.8 to 13.4 mg/L, consisting predominantly of humate and fulvate particles. The dominant anions were bicarbonate and sulfate, at concentrations of 52 and 70 mg/L, respectively. The dominant cations were Na^+^ (up to 47 mg/L) and Ca^2+^ (up to 85 mg/L). Nitrate and phosphate concentrations did not exceed 0.3 and 0.14 mg/L, respectively. Iron and manganese concentrations in the samples were below 2 and 0.3 mg/L, respectively. Thus, the content of biophilic elements in the samples prior to the eutrophication period was low, which is consistent with the classification of this water body as dystrophic.

Trace element analysis and gamma-spectrometric measurements confirmed the absence of technogenic radionuclide contamination. Uranium concentrations were at low, naturally occurring levels typical of surface waters, not exceeding 3 µg/L.

#### 3.1.2. Elemental and Mineralogical Composition of Bottom Sediments

Bottom sediment samples collected prior to the summer bloom period revealed substantial differences in geochemical conditions across the sampling locations. Eh values in samples B1 and B2 were +18 mV, while in sample B3 the value was −76 mV. The bottom sediment samples contain typical rock-forming minerals (Table A2, see Appendix A). Sample B1 is a sandy loam comprising no more than 16% clay minerals. The dominant phase is quartz (71%), accompanied by other rock-forming minerals (plagioclase, K-feldspar, amphibole, and calcite) accounting for up to 13% in total; illite is the predominant phase within the clay fraction, constituting 9%. Sample B2 is a silty loam with a clay fraction content of 33%. Quartz is likewise the dominant phase in this sample (45%), with the remaining rock-forming minerals (plagioclase, K-feldspar, amphibole, and calcite) not exceeding 22% in total; the clay fraction is again dominated by illite (16%). The bottom sediments collected at sampling point 3 consist of quartz and clay minerals (total content 17%), with a high organic matter content (up to 56 wt.%) and pyrite (49%). Remnants of herbaceous vegetation and woody material were present in this sample. The elevated organic matter content in sample B3 is further evidenced by high loss-on-ignition values (Table A2, see Appendix A). Organic matter contents in the remaining samples were comparatively low. The reducing conditions prevailing at point 3 promoted the formation of sulfide-ferrous mineral phases, whereas under the oxidizing conditions characteristic of points 1 and 2, iron content did not exceed 2.25 wt.% expressed as oxide, and sulfur concentrations were below the instrument detection limit. Calcium content likewise indirectly corroborates the presence of calcite or calcium-bearing montmorillonite forms (Table A3, see Appendix A).

### 3.2. Lake Productivity During the Growing Season

Assessment of lake productivity over the 2022 growing season (Table 2) revealed two peaks of phytoplankton development, occurring in mid-July and mid-August, coinciding with increases in mean daily water temperature. The maximum chlorophyll accumulation was recorded in mid-July, reaching 14.6 µg/sample. Chlorophyll concentrations in lake samples collected in April and early October did not exceed 0.1 µg/L. Elevated phytoplankton activity was accompanied by a decline in dissolved oxygen in the water column to 5.1 mg/L. During the autumn and spring periods, dissolved oxygen concentrations ranged from 7.0 to 7.5 mg/L.

### 3.3. Phytoplankton Characterization of Water Samples

**Figure 2:**
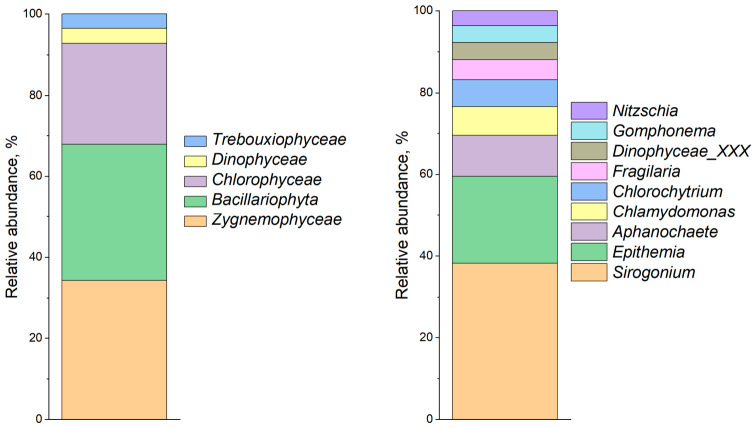
Prokaryotic community diversity profile of the phytoplankton community by 18sRNA genes (family level shown on left, and genus level shown on right).

Taxonomic composition of phytoplankton, assessed by 18S *rRNA* gene analysis (Figure 2), revealed the dominance of diatoms Bacillariophyta (33.16%), green algae *Chlorophyceae* (24.58%), and *Zygnemophyceae* (33.81%) at the phylum level. At the genus level, the dominant taxa were the filamentous alga *Aphanochaete* (7.89%), the unicellular green alga Chlamydomonas (5.61%), the unicellular alga *Chlorochytrium* (5.12%), the diatom *Epithemia* (16.71%), and the filamentous charophyte green alga *Sirogonium* (30.06%).

**Figure 3:**
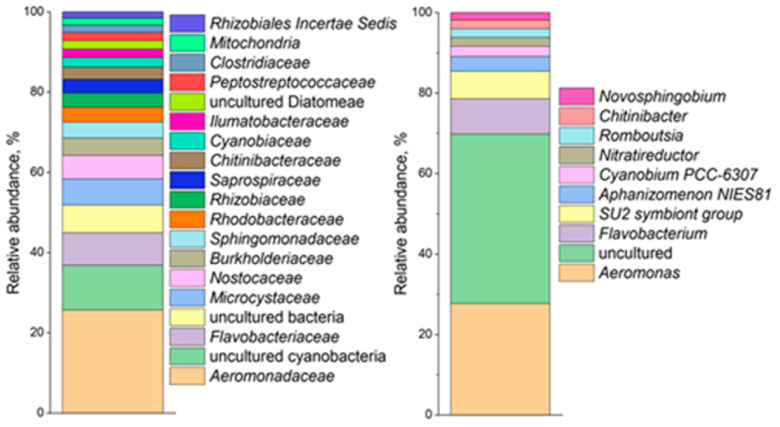
Prokaryotic community diversity profile of the phytoplankton community by 16sRNA genes (family level shown on (**left**), and genus level shown on (**right**)).

Assessment of phytoplankton diversity by 16S rRNA gene analysis (Figure 3) revealed that the dominant taxa at the family level were representatives of Cyanobacteria, including *Microcystaceae* and *Nostocaceae* (up to 35% of OTUs), Gammaproteobacteria including *Aeromonadaceae* (up to 30% of OTUs) and *Chitinibacteraceae* (up to 5% of OTUs), *Bacteroidota* including *Flavobacteriaceae (up to 10% of OTUs)* and *Saprospiraceae* (up to 5% of OTUs), Alphaproteobacteria including *Rhizobiaceae* (up to 5% of OTUs), and Betaproteobacteria including *Burkholderiaceae* (up to 5% of OTUs). At the genus level, the dominant taxa were uncultured cyanobacteria (up to 15%), unicellular diazotrophic cyanobacteria (SU2 symbiont group, up to 10%), filamentous nitrogen-fixing cyanobacteria *Aphanizomenon* (up to 5%), and cyanobacteria *Cyanobium* (up to 5%). Within the domain *Bacteria*, representatives of the genera *Aeromonas* (more than 30%) and *Flavobacterium* (up to 10%) were detected.

### 3.4. Microbial Diversity of Bottom Sediments

**Figure 4:**
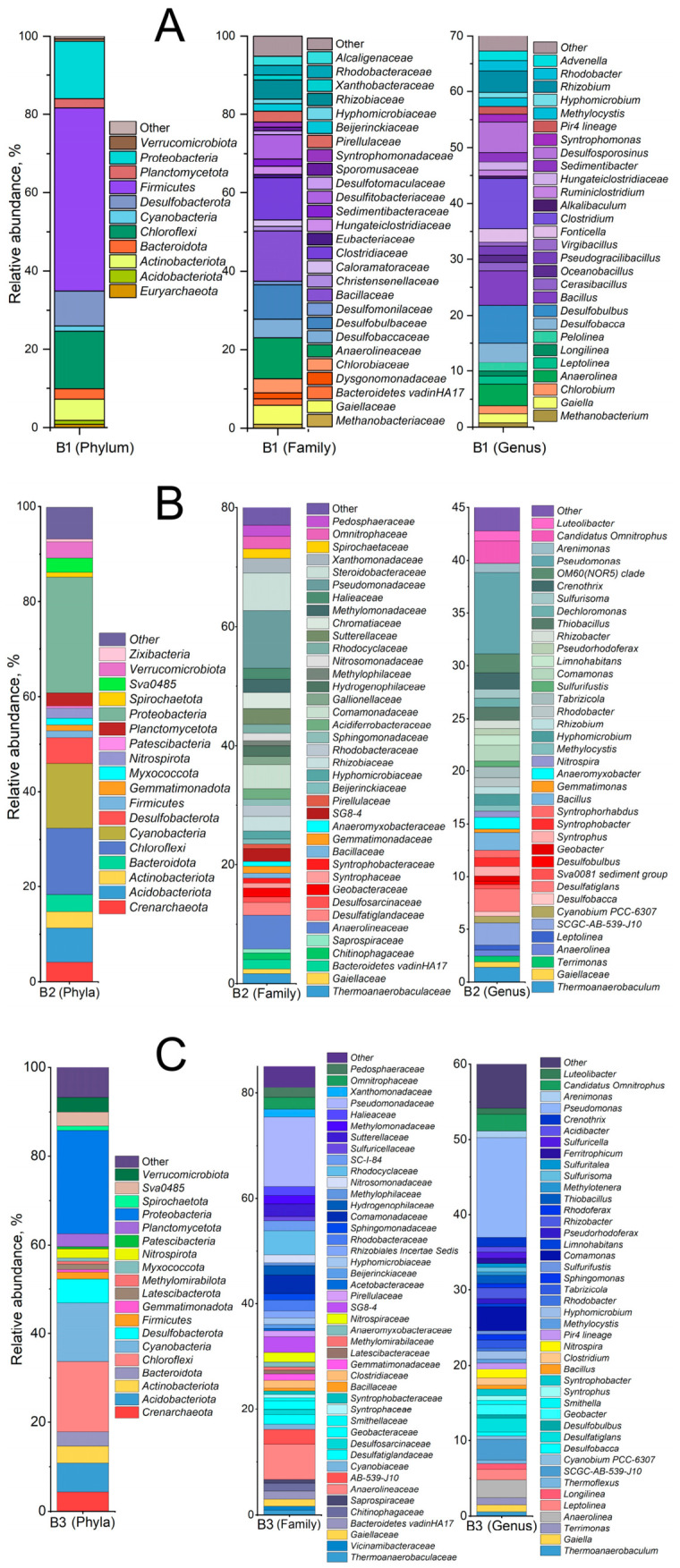
Relative abundances at the phylum, family and genus level within the bacterial community of samples: (**A**) B1, (**B**) B2, (**C**) B3. The abundance was determined from the fragments of bacterial 16S rRNA gene sequences in the libraries, the taxa constituting > 0.5% are listed.

Analysis of the microbial diversity of bottom sediments based on 16S rRNA gene sequencing (Figure 4) revealed a substantial contribution of the phyla *Chloroflexi* and *Proteobacteria* across all sampling sites (>15%). It should be noted that sample B1 exhibited a distinctive community structure, characterized by the dominance of representatives of the phylum *Firmicutes* (46.8%). At the family level, the dominant taxa in community B1 were *Bacillaceae* (12.6%), *Clostridiaceae* (11.0%), *Anaerolineaceae* (10.3%), and *Desulfobulbaceae* (8.5%). Less abundant families included *Desulfitobacteriaceae* (6.0%), *Gaiellaceae* (4.8%), *Rhizobiaceae* (4.8%), and *Chlorobiaceae* (3.5%). Among the minor community members, sample B1 was characterized by the presence of planctomycetes of the family Pirellulaceae (2.8%) and methanogenic archaea (*Methanobacteriaceae*, 1.0%). At the genus level, sample B1 was dominated by *Clostridium* (8.9%), *Desulfobulbus* (6.9%), *Bacillus* (6.1%), *Desulfosporosinus* (5.4%), *Rhizobium* (3.9%), *Anaerolinea* (3.85), *Desulfobacca* (3.5%), *Fonticella* (2.45%), *Chlorobium* (2.0%) and *Methanobacterium* (1.0%).

In community B2, the dominant families were represented by organotrophic bacteria, including *Pseudomonadaceae* (10.2%), *Steroidobacteraceae* (6.2%), *Anaerolineaceae* (5.7%), *Rhodocyclaceae* (4.5%), and *Comamonadaceae* (3.5%). At the genus level, sample B2 was dominated by organotrophic bacteria of the genera *Pseudomonas* (10.2%), *Comamonas* (3.2%), *Dehalococcoidia* (2.8%), *Anaerolinea* (2.3%), *Candidatus Omnitrophus* (2.2%), *Desulfatiglans* (1.8%), and *Rhizobacter* (1.4%). In sample B3, the dominant families were *Pseudomonadaceae* (9.7%), *Steroidobacteraceae* (6.3%), *Anaerolineaceae* (5.6%), and *Comamonadaceae* (4.0%). Representatives of the families *Chromatiaceae* (2.6%), *Sutterellaceae* (2.6%), *Xanthomonadaceae* (2.5%), *Rhizobiaceae* (2.4%), and *Desulfatiglandaceae* (2.1%) were also detected in this sample. At the genus level, sample B3 was dominated by *Pseudomonas* (7.7%), *Dehalococcoidia* (2.1%), *Desulfatiglans* (2.1%), and *Candidatus Omnitrophus* (2.0%), with representatives of the genera *Crenothrix* (1.5%) and *Comamonas* (1.1%) also present.

### 3.5. Laboratory Modeling of Radionuclide Behavior in Water Samples

#### 3.5.1. Chlorophyll Accumulation and Changes in the Composition of Bottom Sediment Samples

**Figure 5:**
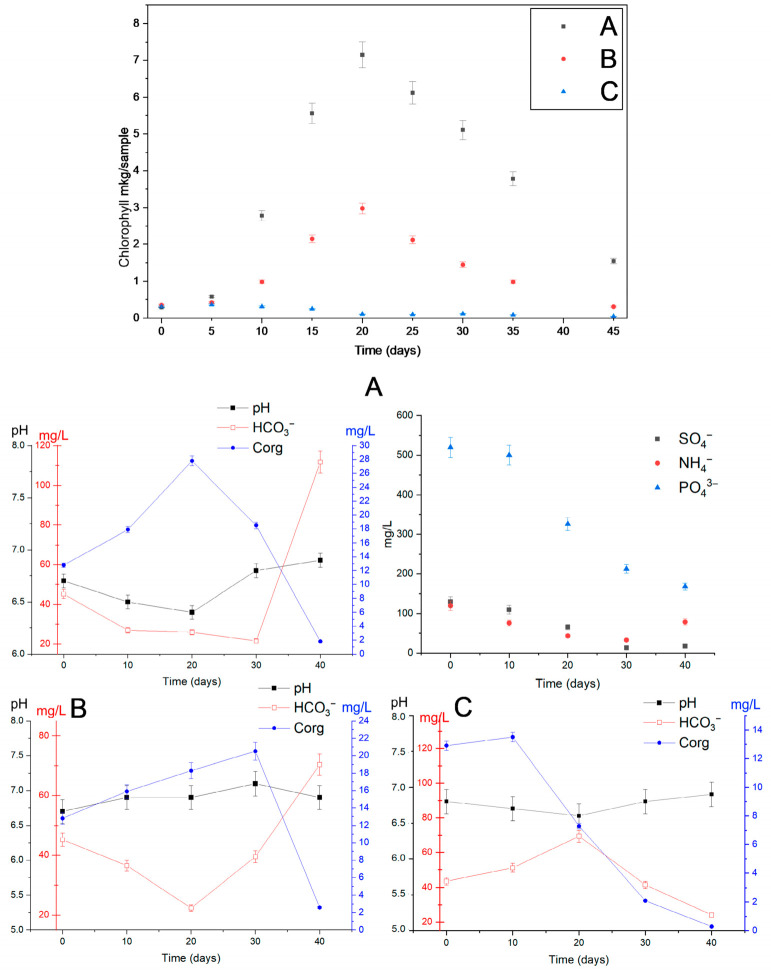
Chlorophyll accumulation (µg per sample) in sample 2 under laboratory experimental conditions at varying trophic levels: (**A**) with phytoplankton activation; (**B**) without phytoplankton activation; (**C**) under dark conditions.

Activation of phytoplankton in sample 2 by Ammophos and sodium sulfate resulted in chlorophyll accumulation, reaching a maximum of 7 µg per sample by day 20 (Figure 5A), after which a decline in chlorophyll content was observed due to cell sedimentation. In the sample without the addition of supplements, the peak of chlorophyll accumulation was likewise recorded on day 20; however, the chlorophyll concentration reached only 3 µg per sample. No chlorophyll accumulation was detected under dark cultivation conditions (Figure 5C).

Throughout the cultivation period, changes in the chemical composition of the samples were observed as a function of cultivation conditions. Under stimulated conditions, consumption of sulfate, ammonium, and phosphate by the phytoplankton community and bottom sediment microorganisms was recorded. Under illuminated conditions, organic matter accumulated to 28 mg/L by days 20–30, depending on the stimulation regime —specifically when phosphorus, sulfur, and nitrogen sources were applied—and to 20mg/L in the absence of activation. By day 40, however, organic matter concentrations declined to 1.8 mg/L under activation conditions and to 2.6 mg/L without activation. Under activation conditions, bicarbonate concentrations decreased most markedly by day 30, followed by an increase to 110 mg/L by day 40. In the non-activated samples, a similar decreasing trend in bicarbonate concentration was observed up to day 20, after which a gradual increase to 70 mg/L was recorded by day 40. In the dark experiment, organic matter consumption proceeded to 0.3 mg/L by day 40, accompanied by an increase in bicarbonate concentration to 69 mg/L by day 20, followed by a subsequent decline to 24 mg/L by day 40.

**Figure 6:**
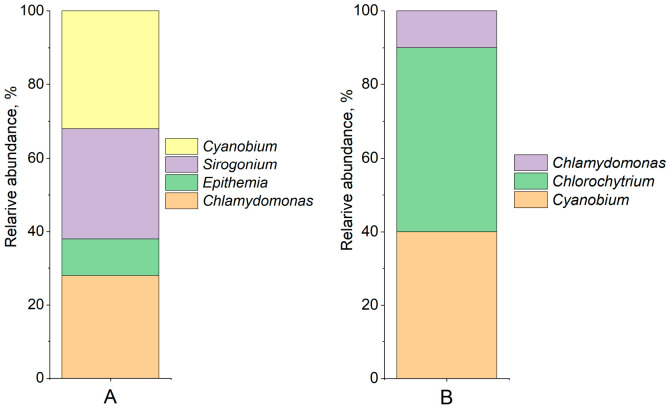
Community diversity of samples after 20 days of cultivation: (**A**) under stimulated conditions; (**B**) without stimulation.

In the sample activated with sulfur, phosphorus, and nitrogen sources, the dominant taxa on day 20 were the unicellular green alga *Chlamydomonas* (28%), the diatom *Epithemia* (10%), the filamentous charophyte green alga *Sirogonium* (30%), and the cyanobacterium *Cyanobium* (32%). In the non-stimulated sample, dominance was recorded for the cyanobacterium *Cyanobium* (40%), the unicellular alga *Chlorochytrium* (50%), and *Chlamydomonas* (10%) (Figure 6A).

On day 20, the activated sample (supplemented with sulfur, phosphorus, and nitrogen sources) was dominated by the unicellular green alga *Chlamydomonas* (28%), the diatom *Epithemia* (10%), the filamentous charophyte *Sirogonium* (30%), and the cyanobacterium *Cyanobium* (32%). In the non-stimulated sample, the dominant taxa were the cyanobacterium *Cyanobium* (40%), the unicellular green alga *Chlorochytrium* (50%), and *Chlamydomonas* (10%) (Figure 6B).

**Figure 7:**
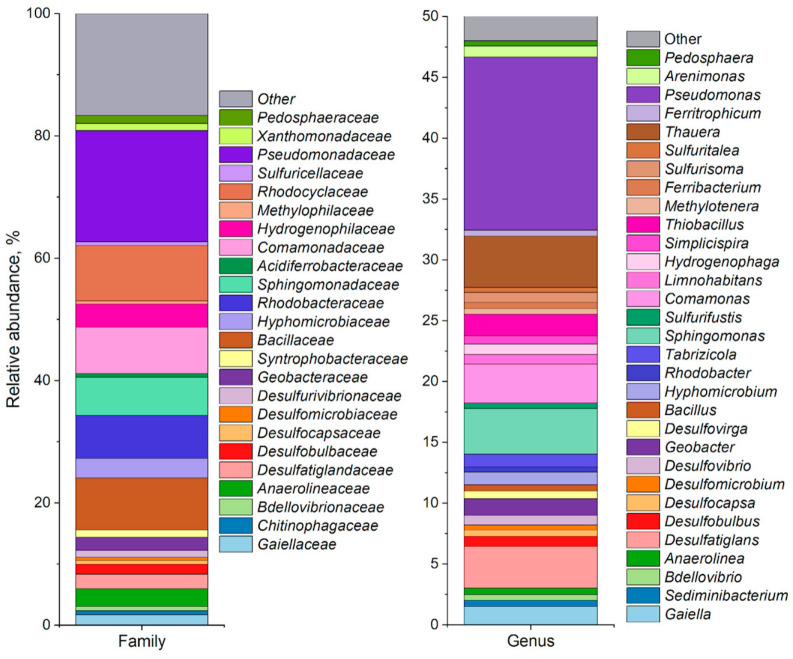
Microbial community composition of lake bottom sediments under laboratory stimulation conditions (at family and genus levels).

In the bottom sediments of sample 2 following activation (Figure 7), representatives of the families *Pseudomonadaceae* (18.2%), *Rhodocyclaceae* (9.0%), *Bacillaceae* (8.5%), *Comamonadaceae* (7.5%), *Rhodobacteraceae* (7.02%), *Sphingomonadaceae* (6.22%), *Hydrogenophilaceae* (3.8%), *Hyphomicrobiaceae* (3.2%), *Anaerolineaceae* (2.9%), *Desulfatiglandaceae* (2.3%), *Geobacteraceae* (2.1%), *Gaiellaceae* (1.7%), *Desulfobulbaceae* (1.6%), and *Desulfurivibrionaceae* (1.13%) were identified. At the genus level, the dominant taxa were *Pseudomonas* (14.2%), *Thauera* (4.2%), *Sphingomonas* (3.7%), *Desulfatiglans* (3.4%), *Comamonas* (3.2%), *Thiobacillus* (1.8%), *Geobacter* (1.38%), *Desulfobulbus* (1.04%), *Sulfurifustis* (1.04%), *Sulfuritalea* (0.9%), *Sulfurisoma* (0.8%), and *Desulfovibrio* (0.8%).

#### 3.5.2. Assessment of Radionuclide Removal from the Aqueous Phase Under Various Experimental Conditions

**Figure 8:**
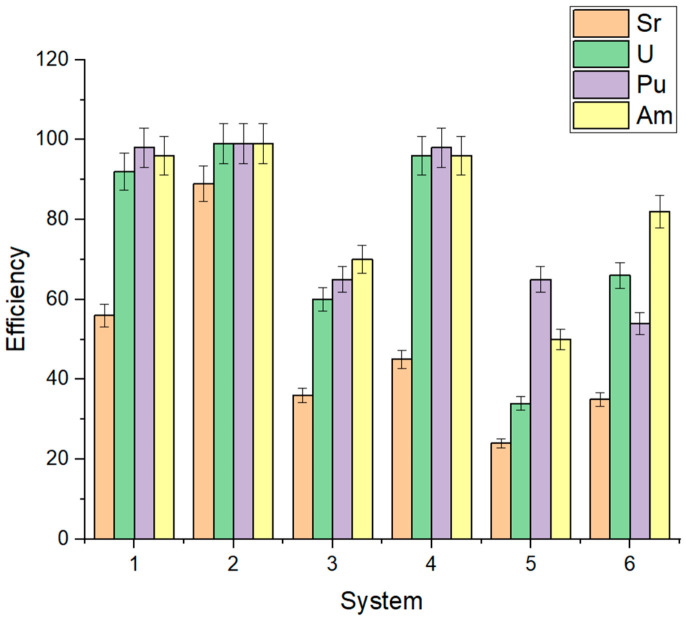
Radionuclide removal efficiency from the liquid phase at day 30: 1—eutrophic conditions, no bottom sediments, light; 2—eutrophic conditions, bottom sediments, light; 3—dystrophic conditions, light; 4—eutrophic conditions, bottom sediments, no light; 5—bottom sediments, no light; 6—sterile water, eutrophic conditions.

Assessment of the removal efficiency of ^90^Sr, ^233^U, ^239^Pu, and ^241^Am (Figure 8) in laboratory experiments demonstrated that under phytoplankton activation conditions without sediment addition (system 1), removal efficiency by day 40 exceeded 96% for Pu and Am, reached 92% for U, and did not exceed 56% for Sr. Under stimulated conditions with sediment addition (system 2), actinide removal efficiency exceeded 99%, while that of Sr reached 79%. Upon addition of bottom sediments from sampling site B2 together with phosphorus, nitrogen, and sulfur sources under dark conditions (system 3), actinide removal efficiency remained high, exceeding 96%, whereas Sr removal did not exceed 45%. The addition of bottom sediments without biophilic elements in the dark (system 4) resulted in the removal of 34% of U, 65% of Pu, 50% of Am, and 24% of Sr from solution. In system 5, consisting of sterile water without sediment supplemented with Ammophos and sodium sulfate, U removal efficiency was 66%, Pu 54%, and Am 82%. In system 6, comprising water without sediment addition under illuminated conditions, U, Pu, and Am removal efficiency reached 60–70% by day 40, while Sr removal did not exceed 36%.

#### 3.5.3. Assessment of Radionuclide Behavior in Bottom Sediments

Assessment of the radionuclide distribution coefficients (K_d_) on bottom sediments (Table 3) revealed considerable differences between samples depending on their composition. Minimum K_d_ values for all radionuclides were observed in the sample consisting predominantly of loamy sand (B1). For Sr, K_d_ values (cm^3^/g) did not exceed 40; for U, 140; for Pu, 170; and for Am, 330. In the sample dominated by the clay fraction (B2), an increase in K_d_ values was observed for all radionuclides relative to sample B1. For Sr, values reached 250; for U, 890; for Pu, 710; and for Am, 1400 cm^3^/g. Maximum K_d_ values for U and Pu (1300 and 9200 cm^3^/g, respectively) were recorded for sample B3, which is dominated by sulfide phases. For Am and Sr, K_d_ values were comparable to those obtained for sample B2. It should be noted that phytoplankton activation in sample 2 resulted in a 3.7-fold increase in K_d_ values for U, a 13-fold increase for Pu, and a 2-fold increase for Am, yielding values comparable to those obtained for sample B3.

**Figure 9:**
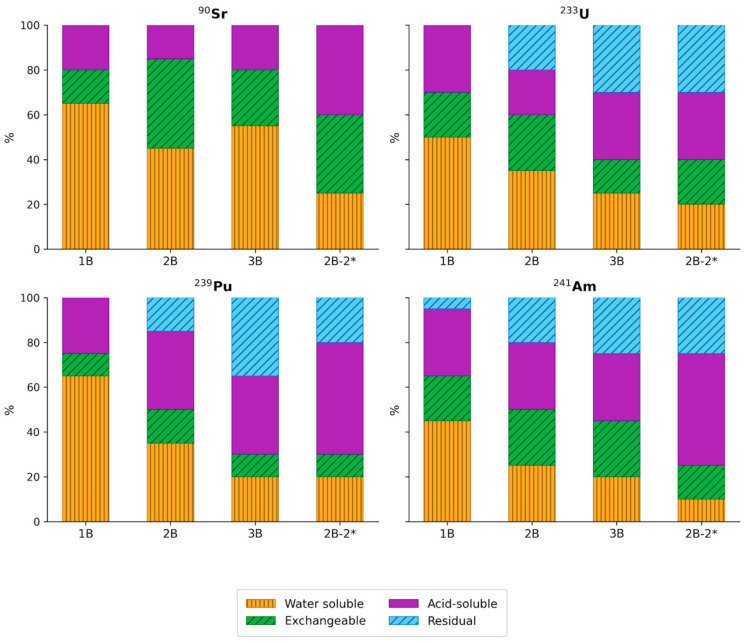
Sequential desorption of radionuclides from bottom sediment samples B1–B3 and sample B2 following stimulation (2B*) (%).

Insights into the mechanisms of radionuclide immobilization are provided, albeit indirectly, by the sequential extraction data (Figure 9). In sample B1, the proportion of weakly bound forms (water-washable and ionic forms extracted with magnesium chloride) accounted for 65–70% of all radionuclides, while the residual fraction was minimal. In sample B2, the proportion of weakly bound forms was 75% for Sr, 60% for U, and 50% for Pu and Am. Furthermore, compared to sample B1, the residual (strongly bound) fraction for actinides accounted for 15–20%. In sample B3, the distribution of Sr between the weakly and strongly bound fractions changed only marginally relative to the other samples, whereas for actinides the proportion of weakly bound forms decreased to 30–40%, and the contribution of the residual fraction increased to 30–35% for U and Pu, and to 25% for Am. In sample B2 following microbial activation, the fractional distribution of Sr changed considerably: the proportion of weakly bound forms decreased from 75 to 45%, while the acid-soluble fraction increased markedly from 15 to 45%. For actinides, a similar decrease in the contribution of weakly bound forms was observed, accompanied by an increase in the proportions of both acid-soluble and strongly bound fractions. For Pu and Am, the most pronounced change was a 20% increase in the contribution of acid-soluble forms.

**Figure 10:**
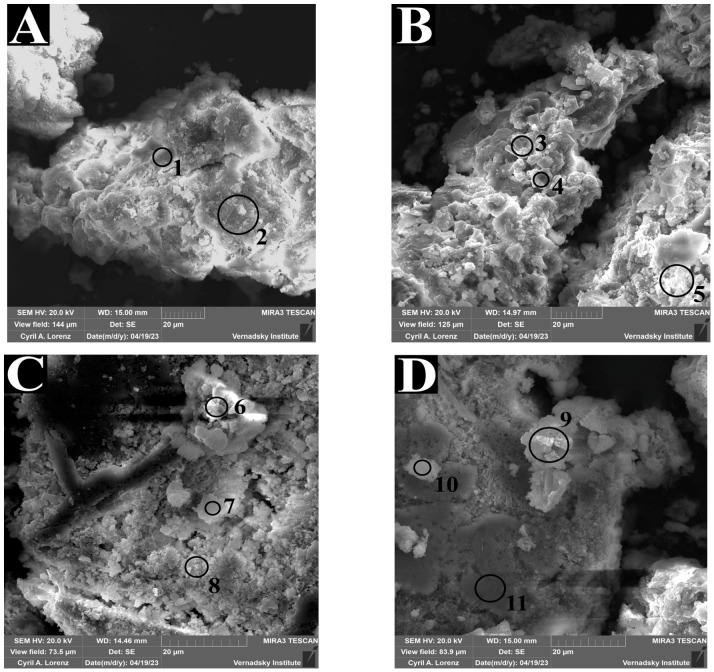
Scanning electron micrographs of bottom sediments from sample B2 before (**A**,**B**) and after microbial stimulation (**C**,**D**). Samples (**A**,**C**): following the addition of uranium (10^−3^ M); samples (**B**,**D**): following the addition of strontium (10^−3^ M). The elemental composition of points 1–11 indicated in the scanning electron micrographs is presented in Table A6.

Scanning electron microscopy analysis of bottom sediments amended with U and Sr (Figure 10, Table A6, see Appendix A) revealed the following. In the experiment without stimulation (Figure 9), mineral phases containing U and Sr were found to be associated predominantly with calcium phases (points 1 and 7 for U; points 3, 4, and 10 for Sr). In addition to calcite phases, experiments conducted with the addition of phosphorus and sulfur sources (Figure 10C,D) revealed accumulation of U and Sr in zones with elevated phosphorus and calcium content (points 6 and 9). At point 8, uranium accumulation was detected in a zone characterized by high Fe and S content.

## 4. Discussion

Lake Dryazlo can be characterized as a low-productivity water body with low concentrations of biophilic elements. The dissolved organic matter content during the spring and autumn periods did not exceed 13 mg/L. Nitrogen and phosphorus concentrations were extremely low. The lake is located in an area with minimal anthropogenic impact, which was further confirmed by the absence of detectable technogenic radionuclides in the water and bottom sediments. Accordingly, this lake represents a suitable model object for investigating the behavior of technogenic radionuclides under natural conditions.

During the period of active bloom in summer, when water temperature exceeds 20 °C, biomass accumulation occurs, with dominance of unicellular (fam. *Chlorophyceae*, *Chlamydomonas*, *Chlorochytrium*) and filamentous (fam. *Aphanochaete*, *Sirogonium*) green algae, diatoms (fam. *Bacillariophyta*, *Epithemia*), and cyanobacteria (gen. *Aphanizomenon*, *Cyanobium*), which are typical of freshwater bodies [41,42,43,44]. This leads to a transient accumulation of organic matter and a decrease in dissolved oxygen concentration to 5.2 mg/L, with dissolved organic matter reaching 22.6 mg/L at peak phytoplankton development. Based on these data, a laboratory experiment was designed to model dystrophic conditions, in which no additional biophilic elements were added to the water samples, and eutrophic conditions, in which nitrogen and phosphorus sources (ammophos) and a sulfur source (sodium sulfate) were introduced.

Assessment of radionuclide concentration reduction in the aqueous phase demonstrated a substantially higher efficiency of radionuclide transfer to bottom sediments compared to experiments conducted in the absence of illumination. The maximum degree of aqueous phase purification from radionuclides was observed under eutrophic conditions, exceeding 98% for actinides and reaching up to 76% for ^90^Sr. This can be attributed to the interaction of actinides and ^90^Sr with phytoplankton biomass and subsequent deposition into bottom sediments following cell sedimentation upon cell death, to the effect of stimulating additives, particularly ammophos, and to changes in physicochemical conditions, such as a decrease in dissolved oxygen content. The latter process may promote the development of reducing conditions, facilitating the reduction of U and Pu to sparingly soluble forms [45]. Following phytoplankton activation, the dominant representatives were the unicellular green alga *Chlamydomonas*, the diatom *Epithemia*, the filamentous charophyte green alga *Sirogonium*, and the cyanobacterium *Cyanobium*. It is well established that radionuclide interactions with cells of these organisms may occur via surface sorption as well as intracellular accumulation. The mechanisms of U accumulation by phytoplankton are described in detail in [46], according to which the U accumulation coefficient for various phytoplankton species ranges from 30–60 to 600–1600. U is sorbed onto the cells themselves and onto their metabolic products, accumulates in the periplasm, and may be reduced by bacterial cells. In [10], using the cyanobacterium *Arthrospira platensis* as a model organism, high uptake and sorption capacity toward ^239^Pu, ^90^Sr, and ^241^Am were demonstrated following the addition of polyphosphates, attributable to biosorption and bioaccumulation processes. *Chlamydomonas* sp. isolated from extreme uranium mine tailings exhibited a U uptake capacity of approximately 4.30 mg U g^−1^ dry biomass via two possible mechanisms: the dominant pathway was biosorption onto cell walls (ca. 90%), with 10% attributable to bioaccumulation [47]. Investigation of the role of cell wall components of the freshwater alga *Chara fragilis* in U sequestration revealed that coprecipitation of uranyl species with CaCO_3_ constitutes the primary binding mechanism, while direct exchange of Ca^2+^ with UO_2_^2+^ plays a minor role [48]. Metabolic processes such as photosynthesis, most likely through pH regulation, play a key role in U uptake by algae. In the green alga *Ankistrodesmus* sp., U is rapidly captured on the cell surface via complexation with carboxylate, amino, and amide groups, which serve as nucleation sites for the precipitation of insoluble rose-like compreignacite (K_2_[(UO_2_)_6_O_4_(OH)_6_]·8H_2_O). Vogel et al. demonstrated that U removal efficiency by living *Chlorella vulgaris* is affected by pH and cell activity [49]. Diatomaceous algae are among the most promising microorganisms for ^90^Sr removal from contaminated water bodies owing to their unique silica-based cell walls (frustules) with abundant silanol groups (Si–OH) that serve as binding sites for divalent cations such as Sr^2+^ [50]. Taking into account phytoplankton biomass productivity, over a single growing season, sorption and subsequent transfer to bottom sediments per gram of biomass may be expected to account for 1.89 × 10^4^ Bq of ^90^Sr, 5.41 × 10^4^ Bq of ^233^U, 6.64 × 10^4^ Bq of ^239^Pu, and 4.04 × 10^4^ Bq of ^241^Am. However, the self-purification of the lake water column through phytoplankton development represents only one aspect of the process; the strength of radionuclide binding within bottom sediments is equally important.

Furthermore, the high radionuclide removal efficiency observed under simulated eutrophic conditions may be attributed to the effect of introduced phosphate on the formation of sparingly soluble compounds with ^90^Sr, U, ^241^Am, and ^239^Pu [51,52,53,54,55]. The contribution of mineral additives is confirmed by the experiment with sterile water (system 6) supplemented with ammophos and sodium sulfate, in which ^90^Sr removal efficiency reached 37%, while that of U, ^241^Am, and ^239^Pu reached 66%, 54%, and 82%, respectively. Thus, based on the results of a laboratory experiment with the addition of ammophos to sterile water, it has been demonstrated that the mechanism of actinide and strontium mineralization in phosphate phases, described in the studies cited above, may play an important role in freshwater bodies upon the addition of a phosphorus source.

Thermodynamic modeling results indicated that under the studied conditions, both chemical and biogenic mineralization of actinides can be expected to occur. Phosphate addition is predicted to promote the formation of the following actinide phases: AmPO_4_(am), autunite, PuO_2_(am), PuPO_4_(s), as well as Sr_3_(PO_4_)_2_(s) (Table A7, see Appendix A). Furthermore, the release of carbon dioxide during the respiration of phytoplankton and microbial communities of bottom sediments may contribute to the formation of sparingly soluble strontium carbonates (strontianite), as well as the inclusion of strontium into minerals of the aragonite group. These processes were described in [56].

U represents the most redox-sensitive element during eutrophication. As a result of a biogenic reduction in the redox potential of the system due to oxygen consumption, U phases, such as uraninite and mixed U(IV) oxides, exhibited a shift in saturation index (SI) toward the formation of less soluble species under both high- and low-trophic conditions [57]. Accordingly, under low-trophic conditions without stimulation, the formation of actinide and ^90^Sr phosphate phases are not predicted. The role of bottom sediment microorganisms in the formation of authigenic mineral phases that facilitate actinide immobilization (sorption and mineralization) is discussed below.

It should be noted that bottom sediments collected from different areas of the lake are heterogeneous in composition: zones with high sand content (sample B1) coexist with zones dominated by clay fractions (sample B2) and zones characterized by organic matter accumulation (sample B3) with localized anaerobic microenvironments and associated sulfide-ferrous mineral phases. Assessment of radionuclide distribution coefficients (K_d_) for bottom sediments (Table 3) revealed substantial differences among samples. Minimum K_d_ values for all radionuclides were recorded for the loamy sand sample (B1). For the clay-dominated sample, K_d_ values increased by a factor of 5–7, while the sulfide-organic sediment sample yielded maximum K_d_ values for U and ^239^Pu (1300 and 9200 cm^3^/g, respectively). Furthermore, assessment of U and ^239^Pu distribution coefficients for sample B2 following microbial activation showed values comparable to those obtained for sample B3, while the K_d_ for ^241^Am exceeded those recorded for sample B3 by nearly a factor of 2. The increase in K_d_ with increasing clay content can be attributed to ^90^Sr sorption by a reversible, non-specific cation exchange mechanism on planar surfaces, whereby ^90^Sr adsorbs as a fully hydrated outer-sphere complex [58,59], and to the formation of various surface complexes for actinides. The retention of actinides on clays (montmorillonite) is governed by a multi-site sorption behavior, which includes cation exchange on planar sites and surface complexation (inner-sphere complexation) on edge sites [60,61]. Clay minerals such as montmorillonite, illite, and kaolinite exhibit a high affinity for uranyl ions through a combination of distinct physicochemical mechanisms involving specific, variable-charge sites located on the edges of the clay platelets, such as aluminol (≡Al–OH) and silanol (≡Si–OH) groups, to which uranyl binds through the formation of inner-sphere surface complexes [62].

The increase in K_d_ for U and ^239^Pu in sample B3 can be attributed to the reducing properties of sulfide ions [63] and ferrous minerals under oxidizing conditions, which form iron oxide coatings [64] that actively immobilize actinides, including ^241^Am [65,66]. The occurrence of redox processes in sample B3 is further supported by the absence of significant differences in ^90^Sr immobilization between samples B2 and B3, which have comparable clay mineral contents.

Sequential desorption data corroborate the conclusions regarding ^90^Sr behavior across the three sediment types. The increased contribution of ion-exchangeable fractions in samples B2 and B3 relative to sample B1 correlates with their higher clay content. The acid-soluble fraction may be associated with the formation of calcium and strontium carbonate mineral phases [67]. For U, the contribution of exchangeable forms increases in sample B2, while in sample B3 the acid-soluble and residual fractions become more prominent. The increase in the acid-soluble fraction can be linked to the role of carbonate minerals, primarily calcite, which is capable of participating in U immobilization, as well as to the contribution of iron-bearing mineral phases. The increase in the residual fraction in sample B3 to 30% may be attributed to the possible microbial reduction of U to sparingly soluble forms. For ^239^Pu, a similar trend is observed across samples B1–B2–B3, differing from U in that iron-bearing fractions play a dominant role in its immobilization in samples B2 and B3. The contribution of exchangeable forms, e.g., on clay minerals, did not exceed 10–15%. For sample B3, approximately 40% of ^239^Pu occurs in strongly bound forms, likely associated with its reduction and immobilization within iron-bearing mineral phases [68,69,70]. For ^241^Am, a similar trend is observed from sample B1 to B3, with a decrease in labile fractions and an increase in exchangeable, acid-soluble, and residual fractions. The residual fractions can be attributed to the presence of iron-bearing minerals, particularly in sample B3, as well as to surface precipitation of Am(OH)_3_ on clay mineral surfaces, for example through the formation of polynuclear hydroxide species at high surface loadings, or incorporation into or association with natural organic matter in aggregated forms [61,71].

The most pronounced changes were observed for sample B2 following microbial activation. The increases in Kd for U by a factor of 3.7, for ^239^Pu by a factor of 13, and for ^241^Am by a factor of 2 can be explained by both chemical and biogeochemical factors. Chemical factors include the formation of phosphate complexes described above, as well as intracellular phosphate accumulation. In our previous studies using the cyanobacterium *Arthrospira* as a model organism, it was demonstrated that accumulation of intracellular polyphosphates can significantly enhance the immobilization of actinides and ^90^Sr [10]. The increase in ^90^Sr accumulation may be related to the expansion of carbonate phases formed through microbial carbon oxidation.

The role of phosphate phases was demonstrated by microanalysis of the precipitate. In all experiments, the formation of calcium-bearing phases was detected (points 1, 3, 4, 7, 10) upon calcite precipitation driven by microbial respiration and CO_2_ release. At points 1 and 7, U, Ca, and O were detected; the co-localization of these elements may indicate partial substitution of Ca^2+^ by uranyl ions in the calcite crystal lattice or uranyl adsorption onto the mineral surface. In experiments with ^90^Sr addition, co-accumulation of Sr, Ca, and O was recorded (points 3, 4, 10), consistent with the precipitation of strontianite (SrCO_3_) or isomorphous substitution of Ca^2+^ by Sr^2+^ in the calcite crystal lattice, yielding a solid solution. In experiments involving the addition of phosphorus and sulfur sources (Figure 10C,D), co-accumulation of P and Ca was detected in addition to calcites (points 6, 9), indicating the formation of calcium phosphate phases. The presence of U and ^90^Sr at these points suggests their co-precipitation with calcium phosphate through incorporation into the crystal lattice, as well as the possible formation of discrete mineral phases such as autunite (hydrated calcium uranyl phosphate) or calcium-strontium phosphate.

Sequential desorption data for sample B2-2 * indicate an increased contribution of the exchangeable and residual fractions for ^90^Sr, likely associated with the formation of strontium phosphate phases or the incorporation of ^90^Sr into iron-bearing phases, for example through inclusion in the structure of biogenic siderite (FeCO_3_) formed during microbial reduction of Fe(III) hydroxides, as well as possible association of ^90^Sr with sulfide minerals (pyrite). For U and other actinides, the most significant changes in desorption mechanisms were observed, manifested as an increase in the acid-soluble and residual fractions. The increase in the residual fraction can be attributed to microbial reduction of U to sparingly soluble forms, which has been described in considerable detail in the literature [72,73,74,75].

The increase in the acid-soluble fraction can be explained primarily by the formation of biogenic iron-bearing mineral phases, arising from the dissolution of pre-existing iron minerals and their redeposition in new forms, in particular sulfide-ferrous phases produced by sulfate-reducing bacteria. Scanning electron microscopy analysis at point 8 revealed co-accumulation of Fe and S, which, given the development of reducing conditions through microbial activity and the low oxygen content at this point, is indicative of iron sulfide formation in the precipitate. This phase may play a significant role in U immobilization, as iron sulfide particles are capable of reducing U(VI) to U(IV), leading to the formation of uraninite. In addition, the formation of freshly precipitated iron hydroxide phases promotes actinide sorption [76].

The transformation of potential iron-bearing phases under conditions of varying trophic status is presented in Table A7 Saturation index values for calcite indicate that its precipitation is more likely under high-trophic than under low-trophic conditions. The formation of Fe(III) hydroxides in bottom sediments is thermodynamically unfavorable; however, other iron mineral phases—goethite, hematite, and magnetite—are predicted to form under all conditions considered. Under high-trophic conditions, the formation of sulfide-ferrous mineral phases (troilite, pyrrhotite) is thermodynamically more favorable.

Analysis of the microbial community of bottom sediments following microbial activation of sample B2 revealed an increased contribution of iron-reducing microorganisms, including representatives of the genera *Pseudomonas*, *Comamonas*, and *Geobacter*, as well as bacteria involved in reductive and oxidative processes of the sulfur cycle, including representatives of the genera *Desulfatiglans*, *Thiobacillus*, *Desulfobulbus*, *Sulfurifustis*, Sulfuritalea, *Sulfurisoma*, and *Desulfovibrio* [77,78,79,80].

**Figure 11:**
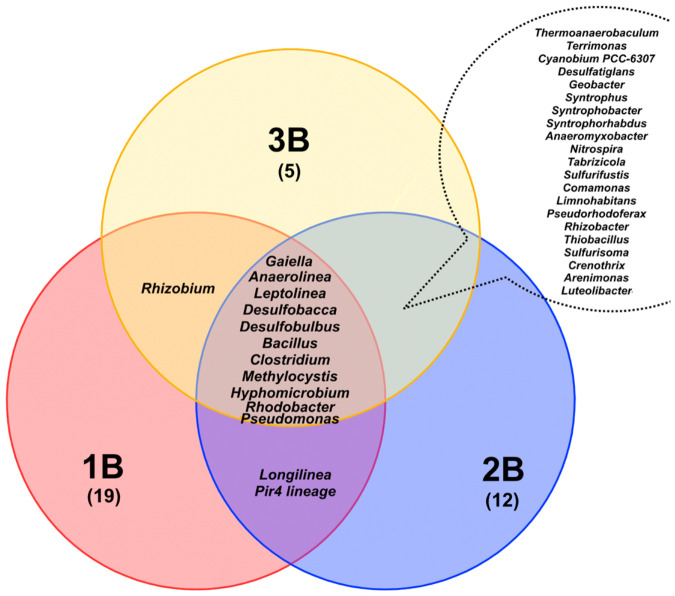
Similarity analysis of sediment and water microbial communities at the genus level (>0.5% relative abundance) (The initial data are presented in the Table A4, See Appendix A).

Considering the lake system as a whole, the microbial communities of the three geochemically distinct sediment samples exhibited broadly similar taxonomic compositions (Figure 11), dominated by aerobic and anaerobic organotrophic bacteria capable of participating in iron–manganese cycles (*Gaiella*, *Pseudomonas*, *Hyphomicrobium*, *Leptolinea*, *Methylocystis*, *Rhodobacter*), sulfur cycles (*Desulfobacca*, *Desulfobulbus*), and carbon cycles (*Anaerolinea*, *Bacillus*, *Clostridium*). It is worth noting that the microorganisms found in the bottom sediment samples are quite typical of freshwater lakes [81,82,83,84].

**Figure 12:**
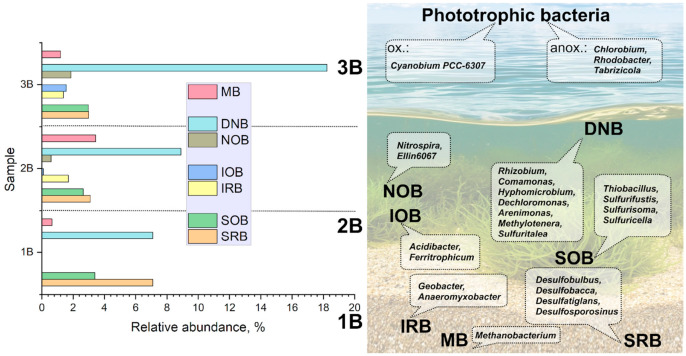
Bacterial physicochemical profiling within water and bottom samples of Lake Dryazlo. MB—methanogenic bacteria/archaea, DNB—denitrifying bacteria, NOB—nitrifying bacteria, IOB—iron oxiding bacteria, IRB—iron reducing bacteria, SOB—sulfur oxiding bacteria, SRB—sulfur reducing bacteria.

The Venn diagram constructed at the genus level reveals considerable overlap between samples B2 and B3, whereas only a single genus of nitrogen-fixing bacteria, *Rhizobium*, is unique to the pair B1 and B3 (Table A4). Microorganisms shared across all communities demonstrate the potential to carry out complete carbon, sulfur, and nitrogen cycling. Despite the high overall similarity between samples B2 and B3, sample B2 exhibits greater diversity, as well as the presence of unique genera of iron- and sulfur-oxidizing bacteria (*Acidibacter*, *Ferritrophicum*, *Sulfuricella*). The community of sample B3 is characterized by the unique presence of bacteria capable of participating in photosynthesis and degrading complex organic matter (*Gemmatimonas*). The bottom sediment community is the most distinct and includes representatives of genera involved in methane production (*Methanobacterium*), obligate anaerobes (*Chlorobium*, *Syntrophomonas*), as well as alkaliphilic bacteria (*Oceanobacillus*, *Virgibacillus*). A number of microorganisms (Figure 12) may participate in photosynthesis and biological nitrogen fixation. Representatives of the genus *Rhodobacter* are capable of anoxygenic photosynthesis and molecular nitrogen fixation [85]. It is noteworthy that denitrifying bacteria belonging to the genera *Hyphomicrobium* and *Pseudomonas* predominate across the samples [86]. Several microorganisms detected in the bottom sediments, including representatives of the genera *Pseudomonas* [87] and *Geobacter* [88], are known for their capacity to participate in the enzymatic reduction of U.

Despite the considerable taxonomic overlap among samples, more pronounced differences in microbial diversity were detected, associated with differences in mineral composition (Table A5). High diversity index values for the microbial communities of the natural lake biome reflect the abundance of OTUs comprising these communities, as well as the absence of clearly dominant taxa in the samples. The bottom sediment community B1, collected from the sand-dominated zone, exhibited the greatest divergence in terms of OTU richness, Chao-1, and Shannon index values. The reduced OTU count and Chao-1 index—which reflects the potential richness of rare and low-abundance species—may indicate a higher degree of community specialization driven by the specific characteristics of this habitat. In contrast, sample B2, characterized by a high clay content, displayed the highest overall diversity as assessed by the Simpson, Shannon, and Chao-1 indices. It is also noteworthy that the Shannon index values for communities B2 and B3 were closely similar, reflecting comparable community structure and evenness. Based on the high Shannon index values, both communities can be characterized as diverse and resilient. The Chao-1 index for community B3 was lower than that for B2, which may indicate a comparatively reduced adaptive potential of this community.

Biogenic mineral formation promoting strong radionuclide binding in bottom sediments likely proceeds at different intensities depending on local physicochemical and geochemical conditions. In summer, high phytoplankton productivity leads to organic matter accumulation in sediments, which may enhance actinide and ^90^Sr mineralization and reduce weakly bound forms. This is expected to be most effective in organic-rich, clay-containing sediments with ferrous sulfide phases, as exemplified by the zone of sample B3. Laboratory experiments confirm that biophilic elements significantly increase phytoplankton productivity and stimulate anaerobic sulfur- and iron-cycling microorganisms in bottom sediments. Thus, adding sulfur, phosphorus, and nitrogen to radionuclide-contaminated lakes may be a promising remediation strategy. These findings agree with previous studies of the Upa River (Chernobyl accident), where nitrogen and phosphorus stimulated *Planktothrix*-dominated phytoplankton, removing Cs, Sr, U, and Pu from water [89]. Enhanced radionuclide immobilization was achieved via sulfide-ferrous precipitates (pyrite, wurtzite, hydrotroilite) formed by sulfate- and iron-reducing bacteria, including *Desulfobacterota*, *Desulfotomaculum*, *Desulfosporomusa*, *Desulfosporosinus*, *Thermodesulfobium*, *Thiomonas*, *Thiobacillus*, *Sulfuritallea*, and *Pseudomonas*.

## 5. Conclusions

Contamination of freshwater bodies may occur as a result of nuclear power plant accidents or nuclear weapon detonations. The behavior of radionuclides in such ecosystems represents a complex problem requiring the consideration of geochemical, biological, and physical factors. Using the dystrophic Lake Dryazlo in the Tver Oblast (Russia) as a model system, the mechanisms of aqueous phase self-purification through phytoplankton development and bottom sediment microbial community activity were analyzed with respect to ^90^Sr, U, ^239^Pu, and ^241^Am. Detailed studies have been carried out for the first time to evaluate the role of phytoplankton and bottom sediment diversity in the removal of actinides and strontium from the water column and their subsequent immobilization in bottom sediments. It was established that, on average over a single growing season under conditions of active phytoplankton biomass development, sorption and subsequent transfer to bottom sediments per gram of biomass—at initial radionuclide concentrations of 82.1 Bq/L for ^90^Sr, 114.7 Bq/L for ^233^U, 153.1 Bq/L for ^239^Pu, and 83.9 Bq/L for ^241^Am—may be expected to amount to 1.89 × 10^4^ Bq of ^90^Sr, 5.41 × 10^4^ Bq of ^233^U, 6.64 × 10^4^ Bq of ^239^Pu, and 4.04 × 10^4^ Bq of ^241^Am. Enhancement of phytoplankton productivity through the introduction of mineral additives is expected to yield a 2–5-fold increase in radionuclide removal efficiency, attributable to the associated increase in phytoplankton biomass. It is important to note that the strength of radionuclide binding following deposition differs substantially depending on the mineral composition of the bottom sediments. The formation of sulfide-ferrous precipitates such as pyrite, wurtzite, and hydrotroilite through the activity of sulfate- and iron-reducing bacteria may lead to a significant increase in the strength of radionuclide immobilization.

Furthermore, the addition of phosphate, nitrogen, and sulfur sources promotes more active phytoplankton development and intensifies the self-purification process, while also facilitating the formation of sparingly soluble phosphate phases of actinides and ^90^Sr. The role of sodium sulfate as a sulfur source was primarily to stimulate sulfate-reducing bacteria in the bottom sediments, thereby facilitating the formation of reduced sulfide phases characteristic of sample B3, which was collected from organically rich sediments. In general, the concentration of sulfate sulfur in the water column was low; therefore, the additional sulfur source, as a biophilic element, plays a role in stimulating phytoplankton through assimilation. Laboratory experiment results indicate that addition phosphorus source itself (as phosphate) promotes the formation of sparingly soluble actinide and strontium phases. The results obtained may form the basis of a bioremediation approach for radionuclide-contaminated freshwater lakes. Increasing the trophic status of a radioactively contaminated water body through the addition of phosphorus and nitrogen sources represents a promising strategy for the conservation and remediation of both natural and engineered radioactive waste storage facilities. The transfer of radionuclides to bottom sediments will clarify the water column, enabling its discharge into the open hydrographic network, while simultaneously establishing an additional biogeochemical barrier within the bottom sediments that prevents radionuclide leaching upon contact with groundwater. It is important to note, however, that eutrophication may induce anoxia, blooms of toxic cyanobacteria, and other undesirable phenomena. Consequently, the proposed strategy is better suited for application primarily in water bodies exhibiting extremely high radionuclide contamination (e.g., waste storage ponds). In the case of natural water bodies with low contamination levels, these potential risks must be taken into account.

## Figures and Tables

**Table 1:** Chemical composition of water samples (mg/L) collected prior to the bloom period.

Sample	L1	L2	L3
pH	6.5 ± 0.25	6.7 ± 0.3	6.5 ± 0.25
C org	13.4 ± 0.1	12.8 ± 0.1	13.2 ± 0.1
TDS	390 ± 5.2	410 ± 5.6	409 ± 6.0
HCO_3_^−^	47.03 ± 0.2	50.12 ± 0.4	52.75 ± 0.5
Cl^−^	4.05 ± 0.005	3.98 ± 0.005	4.23 ± 0.005
NO_3_^−^	0.23 ± 0.001	0.33 ± 0.001	0.31 ± 0.001
SO_4_^2−^	64.84 ± 0.5	69.12 ± 0.5	70.4 ± 0.5
PO_4_^3−^	0.11 ± 0.001	0.14 ± 0.001	0.12 ± 0.001
Na^+^	42.6 ± 0.05	45.8 ± 0.05	47.1 ± 0.05
K^+^	13 ± 0.01	15.5 ± 0.01	14.7 ± 0.01
Mg^2+^	2.93 ± 0.005	3.74 ± 0.005	3.23 ± 0.005
Ca^2+^	85.5 ± 0.5	83.7 ± 0.4	85.9 ± 0.5
Fe	1.9 ± 0.005	1.6 ± 0.005	2.01 ± 0.005
Mn	0.2 ± 0.001	0.3 ± 0.001	0.12 ± 0.001

**Table 2:** Chlorophyll and dissolved oxygen concentrations in water sample 2.

Sampling Date	Chl (µg/Sample)	Water Temperature (°C)	Dissolved O_2_ (mg/L)	C Org (mg/L)
1.04	0.11 ± 0.01	+7 ± 0.1	7.5 ± 0.12	12.8 ± 0.18
15.05	0.22 ± 0.01	+13 ± 0.1	7.3 ± 0.14	13.1 ± 0.11
1.06	0.21 ± 0.01	+16 ± 0.1	7.0 ± 0.15	13.5 ± 0.12
15.06	2.19 ± 0.03	+19 ± 0.1	6.1 ± 0.18	15.4 ± 0.15
1.07	7.31 ± 0.01	+23 ± 0.1	5.5 ± 0.11	19.3 ± 0.10
15.07	14.67 ± 0.5	+22 ± 0.1	5.1 ± 0.10	22.6 ± 0.16
1.08	0.76 ± 0.01	+18 ± 0.1	5.9 ± 0.21	19.7 ± 0.22
15.08	5.75 ± 0.1	+22 ± 0.1	5.3 ± 0.19	21.6 ± 0.21
1.09	0.68 ± 0.03	+15 ± 0.1	5.9 ± 0.12	15.4 ± 0.14
1.10	0.05 ± 0.003	+8 ± 0.1	7.1 ± 0.13	10.6 ± 0.11

**Table 3:** Radionuclide distribution coefficients (K_d_) on bottom sediments (cm^3^/g).

Sample	^90^Sr	^233^U	^239^Pu	^241^Am
B1	0.4 × 10^2^	1.4 × 10^2^	1.7 × 10^2^	3.3 × 10^2^
B2	2.5 × 10^2^	8.9 × 10^2^	7.1 × 10^2^	1.4 × 10^3^
B3	2.2 × 10^2^	3.3 × 10^3^	9.2 × 10^3^	1.8 × 10^3^
B2-2 *	5.2 × 10^2^	3.4 × 10^3^	9.6 × 10^3^	3.9 × 10^3^

* Sediments after activation.

## Data Availability

The original contributions presented in this study are included in the article. Further inquiries can be directed to the corresponding author.

## Abbreviations

The following abbreviations are used in this manuscript:

OTU	operational taxonomic unit
PA	Production Association
PCR	polymerase chain reaction
PHREEQC	pH–REdox–EQuilibrium (C) (geochemical modeling software)
SI	saturation index

## Appendix A

**Table A1:** Sequential extraction applied in this study.

Step	Reagents	Substances Dissolved
1	Distilled water	Water-soluble fraction
2	0.4 M MgCl_2_	Ion-exchangeable fraction
3	1 M HCl	Acid-soluble Fe/Mn oxides

**Table A2:** Mineralogical composition of bottom sediments.

Mineral	B1	B2	B3
Quartz	71	45	37
Plagioclase	8	9	4
K-feldspar	3	10	5
Amphibole	1	2	−
Calcite	1	1	2
Pyrite	−	−	33
Montmorillonite	4	9	12
Illite	9	16	5
Kaolinite	3	8	4

**Table A3:** Elemental composition of bottom sediments (wt. %).

Sample	LOI	Na_2_O	MgO	Al_2_O_3_	SiO_2_	K_2_O	CaO	TiO_2_	MnO	Fe_2_O_3_	P_2_O_5_	S
B1	2.95	2.04	0.68	8.76	79.62	1.75	1.46	0.37	0.04	2.25	0.08	−
B2	6.32	1.97	1.32	9.12	72.01	4.75	1.94	0.10	0.06	2.29	0.12	−
B3	25.87	0.24	0.23	4.53	39.79	1.88	1.30	−	0.02	15.97	−	10.17

**Table A4:** Distribution of taxa in the Venn diagram.

1	10	11	100	101	110	111
Acidaminobacter	Aenigmarchaeales	Desulfomonile	Bdellovibrio	Anaerolinea	Acidibacter	Actinomarinales
Acidaminococcaceae	Altiarchaeaceae	Mycobacterium	Calditrichaceae	Hungateiclostridiaceae	Acidiferrobacteraceae	ADurb.Bin180
Advenella	Candidatus Altiarchaeum	Pelolinea	Flavobacteriaceae		Actinobacteria	Aminicenantales
Aerococcus		Citrifermentans	Flavobacterium		Anaerolineaceae	Afipia
Anaerolineae	Aminicenantales	Curvibacter	Holophagaceae		Alcaligenaceae	GW2011_GWC1_47_15
Anaeromyxobacter	Anaerolineaceae	Dechloromonas	Hydrogenedensaceae		Allorhizobium-Neorhizobium-Pararhizobium-Rhizobium	Haliangium
Anaeromyxobacteraceae	Armatimonadota	Gracilibacteria	Lautropia		Anaeromusa-Anaeroarcus	Halioglobus
Arenimonas	Bacillus	Haliangium	Rhodoferax		Anaerosalibacter	Holophagae
Bacteroidetes BD2-2	Bacteroidetes vadinHA17	Halioglobus	S-BQ2-57 soil group		Anaerovorax	Ignavibacteria
Bathyarchaeia	Beijerinckiaceae	Holophagae	SWB02		Arenicellaceae	Leptothrix
BSV26	Chloroplast	Ignavibacteria	Tropicimonas		Babeliales	Limnohabitans
Candidatus Omnitrophus	Desulfobacca	Leptothrix	Verrucomicrobiaceae		Bacillaceae	Methylomonas
CCM19a	Desulfobaccaceae	Limnohabitans			Bauldia	Nitrospira
Chitinophagaceae	Desulfobacterota	Methylomonas			Bosea	Novosphingobium
Comamonadaceae	Desulfobulbaceae	Nitrospira			Butyricicoccus	P3OB-42
Comamonas	Desulfobulbus	Novosphingobium			Caldisericum	Polymorphobacter
Conexibacter	Gaiella	P3OB-42			Caloramatoraceae	Solibacillus
Crenarchaeota	Gaiellaceae	Polymorphobacter			Candidatus Woesebacteria	Subgroup 10
Crenothrix	Gaiellales	Solibacillus			Caproiciproducens	Subgroup 7
Cyanobiaceae	Hyphomicrobiaceae	Subgroup 10			Cerasibacillus	Sulfuricurvum
Cyanobium PCC-6307	Hyphomicrobium	Subgroup 7			Chlorobaculum	Sulfurimonadaceae
Dehalococcoidia	KD4-96	Sulfuricurvum			Chlorobiaceae	TRA3-20
Desulfatiglandaceae	Leptolinea	Sulfurimonadaceae			Chlorobium	vadinHA49
Desulfatiglans	Longilinea	TRA3-20			Christensenellaceae	
Desulfosarcinaceae	Luteolibacter	vadinHA49			Christensenellaceae R-7 group	
Dinghuibacter	MB-A2-108				Clostridiaceae	
Ellin6067	Methylocystis				Corynebacterium	
Frankiales	Microtrichales				Desulfitobacteriaceae	
FW22	OPB41				Desulfitobacterium	
Gemmatimonadaceae	PeM15				Desulfohalotomaculum	
Gemmatimonas	Pir4 lineage				Desulfomonilaceae	
Geobacter	Pirellulaceae				Desulforhabdus	
Geobacteraceae	Pseudomonas				Desulfosporosinus	
GIF3	Rhizobiales Incertae Sedis				Desulfotomaculaceae	
GIF9	Rhodobacter				Desulfotomaculum	
Halieaceae	Rhodobacteraceae				Devosia	
Hydrogenophilaceae	SC-I-84				Dysgonomonadaceae	
JG30-KF-CM66	SJA-15				Fonticella	
Latescibacteraceae	Solirubrobacterales 67-14				Fusibacter	
Latescibacterota	Steroidobacteraceae				Gastranaerophilales	
LCP-89	Subgroup 17				Gracilibacteraceae	
Lentimicrobiaceae	Sva0485				Herbinix	
MBNT15	Syntrophobacter				IMCC26207	
Methylomonadaceae	Syntrophobacteraceae				Irregularibacter	
Methylophilaceae	Syntrophobacterales				Lachnospiraceae	
Methylotenera	Syntrophorhabdus				LD1-PB3	
MSB-5B2	Thermodesulfovibrionia				Leucobacter	
MSBL5	WCHB1-41				Levilinea	
Napoli-4B-65	WCHB1-81				Lutispora	
NB1-j					Macellibacteroides	
Nitrosomonadaceae					Mesorhizobium	
Nitrospirota					Methanobacteriaceae	
OM190					Methanobacterium	
OM60(NOR5) clade					Methanomicrobiales	
Omnitrophaceae					Methanosarcina	
P9 × 2b3D02					Methylobacter	
Pedosphaeraceae					Myxococcaceae	
Phycisphaerales					NK4A214 group	
Pseudomonadaceae					Oceanobacillus	
Pseudorhodoferax					Oxobacter	
RBG-13-54-9					Paracoccus	
RBG-16-58-14					Pelosinus	
Rhizobacter					Pelotomaculum	
Rhodocyclaceae					Peptococcaceae	
Roseomonas					Petrimonas	
Rubritaleaceae					Prolixibacteraceae	
S085					Propionibacteriaceae	
Saprospiraceae					Pseudogracilibacillus	
SAR324 clade (Marine group B)					Raoultibacter	
SCGC-AB-539-J10					RBG-16-55-12	
SG8-4					Rhizobiaceae	
SM1A02					Sandaracinaceae	
Smithella					SBR1031	
Solirubrobacteraceae					Sedimentibacter	
Sphingomonadaceae					Sedimentibacteraceae	
Spirochaetaceae					Sericytochromatia	
Sporichthya					Spirochaeta	
Subgroup 18					Sporacetigenium	
Subgroup 22					Sporomusaceae	
Sulfurifustis					SRB2	
Sulfurisoma					Syntrophomonadaceae	
Sutterellaceae					Syntrophomonas	
Sva0081 sediment group					Tepidimicrobium	
Syntrophales					Tissierella	
Syntrophus					Trichococcus	
t0.6.f					UCG-010	
Tabrizicola					Verticiella	
Terrimonas					Virgibacillus	
Thermoanaerobaculaceae					WPS-2	
Thermoanaerobaculum					WS1	
Thermoflexus					Xanthobacteraceae	
Thermoplasmata						
Thiobacillus						
vadinBA26						
Vicinamibacteraceae						
Vicinamibacterales						
Vicinamibacteria						
Woesearchaeales						
Xanthomonadaceae						
Zixibacteria						

**Table A5:** Diversity indices of bottom sediment samples.

Index	B1	B2	B3
OTU	310	620	548
Simpson_1-D	0.97	0.97	0.97
Shannon H	4.53	4.88	4.82
Chao-1	460.6	880.9	805

**Table A6:** Elemental composition of points shown in Figure 10 (wt. %).

Point	O	Na	Mg	Al	Si	P	S	Ca	Fe	Sr	U
1	65.75	1.09	0.79	0.74	0.98	−	−	26.76	−	−	3.89
2	54.28	0.67	1.57	10.48	28.24	−	−	1.90	0.98	−	1.88
3	67.25	0.11	0.15	0.42	0.84	−	−	29.84	−	1.39	−
4	61.17	0.21	0.42	0.12	0.29	−	−	34.33	−	3.46	−
5	58.57	1.23	1.72	8.97	26.25	−	−	2.63	−	0.63	−
6	57.81	0.37	1.01	1.35	1.92	12.43	2.34	17.74	1.90	−	3.14
7	69.30	0.80	1.11	1.82	2.81	0.63	−	21.54	−	−	1.99
8	36.92	0.34	0.69	2.15	5.51	1.59	28.13	2.86	21.81		
9	59.97	0.11	0.10	0.79	1.25	9.91	1.28	24.21	0.41	1.97	
10	63.10		0.3	0.13	0.29			33.51	0.31		2.36
11	55.97	1.41	0.98	9.30	30.43			1.13		0.78	

**Table A7:** Saturation index (SI) heatmap of radionuclide mineral phases and main mineral phases during phytoplankton development under high-trophy (HT) and low-trophy (LT) conditions.

Radionuclide Mineral Phases		
Mineral Phase	HT	LT
Autunite	2.7	−1.2
Uraninite	3.2	1.1
UO_2_am	1.5	0.92
AmPO_4_	4.9	−3.2
PuPO_4_	6.2	−4.8
PuO_2_am	3.7	1.1
Ningyoite	4.5	−1.2
Strontianite	1.3	0.98
Sr_3_(PO_4_)_2_	2.4	−3.3
**Main Mineral Phases**		
**Mineral Phase**	**HT**	**LT**
Calcite	2.5	0.9
Fe(OH)_3_	−2.5	−0.4
Goethite	0.7	1.5
Hematite	1.2	1.8
Magnetite	0.8	1.4
Troilite	2.6	1.1
Pyrrhotite	3.2	1.8
